# Coupling wastewater‐based epidemiology with data‐driven machine learning for managing public health risks

**DOI:** 10.1111/risa.70075

**Published:** 2025-07-06

**Authors:** Sheree Pagsuyoin, Calvin Ng, Nerissa Molejon, Yan Luo

**Affiliations:** ^1^ Department of Civil and Environmental Engineering University of Massachusetts Lowell Lowell Massachusetts USA; ^2^ Department of Electrical and Computer Engineering University of Massachusetts Lowell Lowell Massachusetts USA

**Keywords:** infectious diseases, machine learning, predictive analytics, public health, wastewater‐based epidemiology

## Abstract

Traditional health surveillance methods play a critical role in public health safety but are limited by the data collection speed, coverage, and resource requirements. Wastewater‐based epidemiology (WBE) has emerged as a cost‐effective and rapid tool for detecting infectious diseases through sewage analysis of disease biomarkers. Recent advances in big data analytics have enhanced public health monitoring by enabling predictive modeling and early risk detection. This paper explores the application of machine learning (ML) in WBE data analytics, with a focus on infectious disease surveillance and forecasting. We highlight the advantages of ML‐driven WBE prediction models, including their ability to process multimodal data, predict disease trends, and evaluate policy impacts through scenario simulations. We also examine challenges such as data quality, model interpretability, and integration with existing public health infrastructure. The integration of ML WBE data analytics enables rapid health data collection, analysis, and interpretation that are not feasible in current surveillance approaches. By leveraging ML and WBE, decision makers can reduce cognitive biases and enhance data‐driven responses to public health threats. As global health risks evolve, the synergy between WBE, ML, and data‐driven decision‐making holds significant potential for improving public health outcomes.

## 1 INTRODUCTION

Disease surveillance is a cornerstone of public health safety, particularly as both recurring and emerging threats continue to challenge global healthcare systems (Arthur et al., 2011). Effective surveillance is essential in addressing the complex and evolving nature of public health risks, which can be influenced by disease transmission dynamics, environmental factors, and lifestyle behaviors (Eisenberg et al., 2007; Schermer et al., 2022). For instance, during the recent COVID‐19 (Coronavirus Disease 2019) pandemic, rapid health data collection enabled swift decision‐making and timely interventions to curb transmission (Lipsitch et al., 2024). Consistent surveillance is also essential for tracking seasonal diseases like influenza and measles, enabling healthcare professionals prepare for surges in cases (Feldblyum & Segal, 2015; Grassly & Fraser, 2006). While short‐term surveillance is key in managing acute health conditions (e.g., respiratory syncytial virus or RSV; Centers for Disease Control and Prevention, 2024b), long‐term surveillance is necessary for managing chronic diseases (e.g., diabetes, heart disease, and stroke; Centers for Disease Control and Prevention, CDC, 2024a) that are influenced by environmental or lifestyle factors.

In public health monitoring, the role of the health decision maker can be viewed through the lens of Kahneman's concept of “thinking, fast and slow,” which distinguishes between rapid, experience‐based decisions (“fast thinking”) and deliberate, analytical decision‐making (“slow” thinking). Kahneman argues that human decisions are often influenced by cognitive biases and heuristics, which can lead to systematic errors in judgment (Kahneman, 2011). In health decision‐making, these biases may manifest in the over‐reliance on intuition or past experiences rather than fully leveraging available clinical or environmental data. By integrating comprehensive data analysis into decision‐making processes, health officials can better navigate these cognitive pitfalls, ensuring more evidence‐based and effective responses to public health risks (Michel, 2020).

In recent years, big data analytics has become an invaluable tool for understanding public health challenges such as disease transmission and health lifestyle trends. Data‐driven diagnostic and predictive models have been instrumental in analyzing infectious diseases transmission patterns (Skrip & Townsend, 2019), detecting emerging health crises (Dixon et al., 2024), and assessing the long‐term effects of lifestyle changes (Pearson et al., 2020). For instance, large datasets from epidemiological, environmental, and mobility surveillance have been used to track COVID‐19 wave trends (Pagsuyoin et al., 2022) and estimate infection rates under various intervention scenarios (Schenk et al., 2024). Similarly, health lifestyle models that monitor trends in obesity (Vineetha et al., 2024), diabetes (Nandan et al., 2024), and other lifestyle‐related diseases (Ren et al., 2023) can help identify at‐risk populations and inform targeted interventions to mitigate these risks.

Recently, wastewater‐based epidemiology (WBE) has gained increased scientific interest as a public health surveillance tool via the detection of human biochemical markers in wastewater (Bowes et al., 2024). WBE enables near real‐time surveillance of disease prevalence, enabling public health authorities to respond swiftly and effectively to emerging outbreaks (Brosky et al., 2024; Eaton et al., 2022). For example, during the COVID‐19 pandemic, tracking RNA residues of the SARS‐CoV‐2 (severe acute respiratory syndrome coronavirus‐2) virus in wastewater provided an early warning system for rising case numbers in affected areas (Ai et al., 2021; Schenk et al., 2024).

Integrating WBE with machine learning (ML) has the potential to significantly improve predictive health decision‐making. Multi‐modal ML algorithms can process vast amounts of environmental data collected through wastewater sampling, environmental sensors, and other sources to identify disease trends and forecast future public health risks. By training models on multi‐modal datasets, ML can discover patterns in disease spread, social behaviors, and environmental changes that could influence health outcomes. This predictive capability enables more proactive decision‐making and resource allocation, improving our ability to manage public health threats. Coupling WBE with ML can provide decision makers with a more comprehensive view of public health status, enabling targeted interventions and more effective health risks management (Lai et al., 2023; Zehnder et al., 2023). As public health risks evolve, the demand for timely, data‐driven decision‐making underscores the importance of innovative technologies like WBE and predictive analytics. Together, these tools can help mitigate public health threats more efficiently, ultimately improving health outcomes in an increasingly resource‐constrained healthcare system.

This paper explores the current state of science in data‐driven ML approaches for WBE data analytics. We examine their capabilities and propose new use scenarios for ML‐WBE models. Specifically, we advocate for the development of “what‐if” scenarios in health policy modeling, where trained ML models can be leveraged to evaluate the potential impacts of various policy decisions on community wellbeing. We propose a novel data‐driven ML and trend forecasting framework that incorporates historical epidemiological data with hypothetical simulated policy decisions into trained ML models in order to explore and contrast the impacts of different interventions. We also propose a prospect theory‐based 2‐stage data‐driven decision‐making framework that integrates ML models to help inform decisions for public health. We describe how ML models can contribute to both the editing phase and evaluation phase of the model development, particularly as an added input that is free from human biases. This approach enhances the utility of ML models by proactively assessing the consequences of different decision strategies.

The remainder of the paper is organized as follows: Section 2 provides an overview of WBE and its applications in infectious disease monitoring, Section 3 explores ML‐driven WBE modeling for health forecasting. Section 4 discusses our perspectives on the key challenges associated with ML‐WBE models in public health surveillance. Finally, Section 5 outlines current limitations and opportunities for future research in this emerging field.

## 2 WASTEWATER‐BASED SURVEILLANCE OF INFECTIOUS DISEASES

The concept of *WBE* is derived from principles of material flow balance: Sewage samples are analyzed for target chemicals or biomarkers whose measured concentrations are used to back‐calculate community drug consumption or disease infection rates. WBE was first proposed in the United States originally for pharmaceuticals in human medication (Daughton, 2001) and has since been refined and applied extensively to analyze in‐country and inter‐country consumption patterns for various illicit drugs (Luo et al., 2023; Mendoza et al., 2014; van Nuijs et al., 2011; Zuccato et al., 2008). While WBE theory has been around for nearly two decades, it did not gain significant global traction until the recent COVID‐19 pandemic. In the early stages of the pandemic when patient testing was very limited, countries turned to sewage detection of SARS‐CoV‐2 RNA to track infection hotspots (Medema et al., 2020). Fueled by greater scientific and public interest and substantial funding infusion, WBE research—mainly for COVID and more recently for other infectious diseases—has advanced significantly in the last 5 years. Upstream sewage sampling is now feasible (Harris‐Lovett et al., 2021), and devices that simplify sewage processing are commercially available (Zheng et al., 2022). Centralized public reporting of wastewater data is powered by sophisticated data analytics, for example, the US CDC's National Wastewater Surveillance System houses wastewater data from over 1500 sampling locations representing 151 M people (as of January 31, 2025; Centers for Disease Control and Prevention, 2025). Interestingly, while WBE for drug use surveillance predates COVID surveillance, it lags in the magnitude of implementation. Literature on COVID surveillance in the last 5 years is significantly more abundant than literature on drug surveillance in the last 20 years.

In WBE infectious disease surveillance, the process begins with sewage sampling at the inlet lines of wastewater facilities or from building clean‐outs or sewer manholes (Harris‐Lovett et al., 2021). These samples require on‐site preservation immediately after sampling and while in transit to laboratories, usually via storage in cooled containers, to prevent the genetic material from degrading. Upon receipt at the laboratories, the samples are processed using molecular techniques such as quantitative polymerase chain reaction (PCR) and next‐generation sequencing to extract and quantify viral or bacterial biomarkers. Genomic data from these analyses are then integrated with epidemiological data to determine infection rates and inform intervention decisions.

Because sewage can be sampled any time and analyzed for as many biochemical markers as desired, WBE is rapid and cost‐effective (Luo et al., 2023). It can complement traditional health surveillance by offering greater flexibility and efficiency in acquiring health data from sub‐populations at desired timescales. It has the capability to identify evolving patterns of diseases and provide an early warning for emerging diseases (Singer et al., 2023). Global interest in WBE has led to coordinated platforms for best practices in sewage testing and joint surveillance of highly infectious diseases, including re‐merging diseases such as polio (Grassly et al., 2025; Mao et al., 2020). Table 1 summarizes some of recent and on‐going large‐scale coordinated WBE surveillance projects for infectious diseases worldwide. The monitored pathogens include highly infectious respiratory viruses such as SARS‐CoV‐2, influenza A, and RSV, and non‐respiratory pathogens such as norovirus, rotavirus, and hepatitis A. Sampling frequencies vary from daily to monthly. The geographical scope of sampling ranges from citywide initiatives to multi‐country networks.

**TABLE 1:** Global wastewater‐based surveillance of infectious diseases.

Pathogen of interest	Geographical scope	Sampling frequency	Surveillance duration^a^	Reference
*Candida auris*	The United States (nationwide)	Every 2 days	∼18 months (ongoing)	Boehm et al. (2024)
Enterovirus D68	The United States (nationwide)	Every 2 days	∼16 months (ongoing)	Boehm et al. (2024)
Hepatitis A virus	The United States (nationwide)	Daily	∼18 months (ongoing)	Boehm et al. (2024)
	Yongin, South Korea (citywide)	Twice a month	1 year (12/2022–11/2023)	Kim et al. (2024)
Human metapneumovirus	United States (nationwide)	Daily	∼30 months (ongoing)	Boehm et al. (2024)
Influenza A virus	United States (nationwide)	Weekly	∼29 months (ongoing)	Kirby et al. (2021)
	Italy (nationwide)	Monthly	7 months (10/2022–4/2023)	Mancini et al. (2024)
H5 variant (avian)	The United States (nationwide)	Weekly	∼8 months (ongoing)	Boehm et al. (2024)
Influenza B	The United States (nationwide)	Daily	∼22 months (ongoing)	Boehm et al. (2024)
	Italy (nationwide)	Monthly	7 months (10/2022–4/2023)	Mancini et al. (2024)
Human parainfluenza virus	The United States (nationwide)	Every 2 to 3 days	∼6 months (10/2023–5/2024)	Boehm et al. (2024)
Measles	Yongin, South Korea (citywide)	Twice a month	1 year (12/2022–11/2023)	Kim et al. (2024)
Monkeypox virus (mpox)	The United States (nationwide)	4‐week rolling	∼30 months (ongoing)	Boehm et al. (2024)
	Yongin, South Korea (citywide)	Twice a month	1 year (12/2022–11/2023)	Kim et al. (2024)
Norovirus	The United States (nationwide)	Daily	∼26 months (ongoing)	Boehm et al. (2024)
Poliovirus	Yongin, South Korea (citywide)	Twice a month	1 year (12/2022–11/2023)	Kim et al. (2024)
Rotavirus	The United States (nationwide)	Daily	∼15 months (9/2023–12/2024)	Boehm et al. (2024)
Respiratory syncytial virus	The United States (nationwide)	Daily	∼38 months (ongoing)	Boehm et al. (2024)
	The United States (nationwide)	Weekly	∼34 months (ongoing)	Kirby et al. (2021)
SARS‐CoV‐2	The United States (nationwide)	Weekly	∼5 years (ongoing)	Kirby et al. (2021)
	Attica, Greece (municipality‐wide)	Daily	∼7 months (9/2020–3/2021)	Galani et al. (2022)
	Worldwide (185 countries and territories)	Daily	3 years (1/ 2020–12/2022)	Hale et al. (2023)
	Yongin, South Korea (citywide)	Twice a month	1 year (12/2022–11/2023	Kim et al. (2024)
	Catalonia, Spain (regionwide)	Weekly or bi‐weekly	∼2 years (7/2020–3/2022)	Guerrero‐Latorre et al. (2022)
	Bangkok, Thailand (citywide)	Bi‐monthly	5 months (1/2023–5/2023)	Saita et al. (2025)
	Ontario, Canada (provincewide)	2–7x a week	∼2 years (1/2021–3/2023)	D'Aoust et al. (2024)
Zika virus	Yongin, South Korea (citywide)	Twice a month	1 year (12/2022–11/2023)	Kim et al. (2024)

^a^ Data as of January 31, 2025.

Since its application in WBE COVID‐19 surveillance, molecular techniques have remained as the gold standard in detecting disease pathogens in wastewater worldwide (Wu et al., 2021). However, testing efforts can be slowed down by bottlenecks in the supply chain (e.g., shortage in RNA kits) and by time‐consuming laboratory analysis. Furthermore, existing PCR‐based testing requires highly skilled technicians and well‐equipped laboratories, presenting challenges for rapid on‐site mass testing in communities (Dien Bard & Babady, 2022). Advanced analytical methods, including ML and predictive modeling, can further enhance the interpretation of WBE data, enabling early warning systems for outbreaks and facilitating data‐driven decision‐making for public health authorities.

## 3 DATA‐DRIVEN ML FOR PUBLIC HEALTH FORECASTING

**FIGURE 1:**
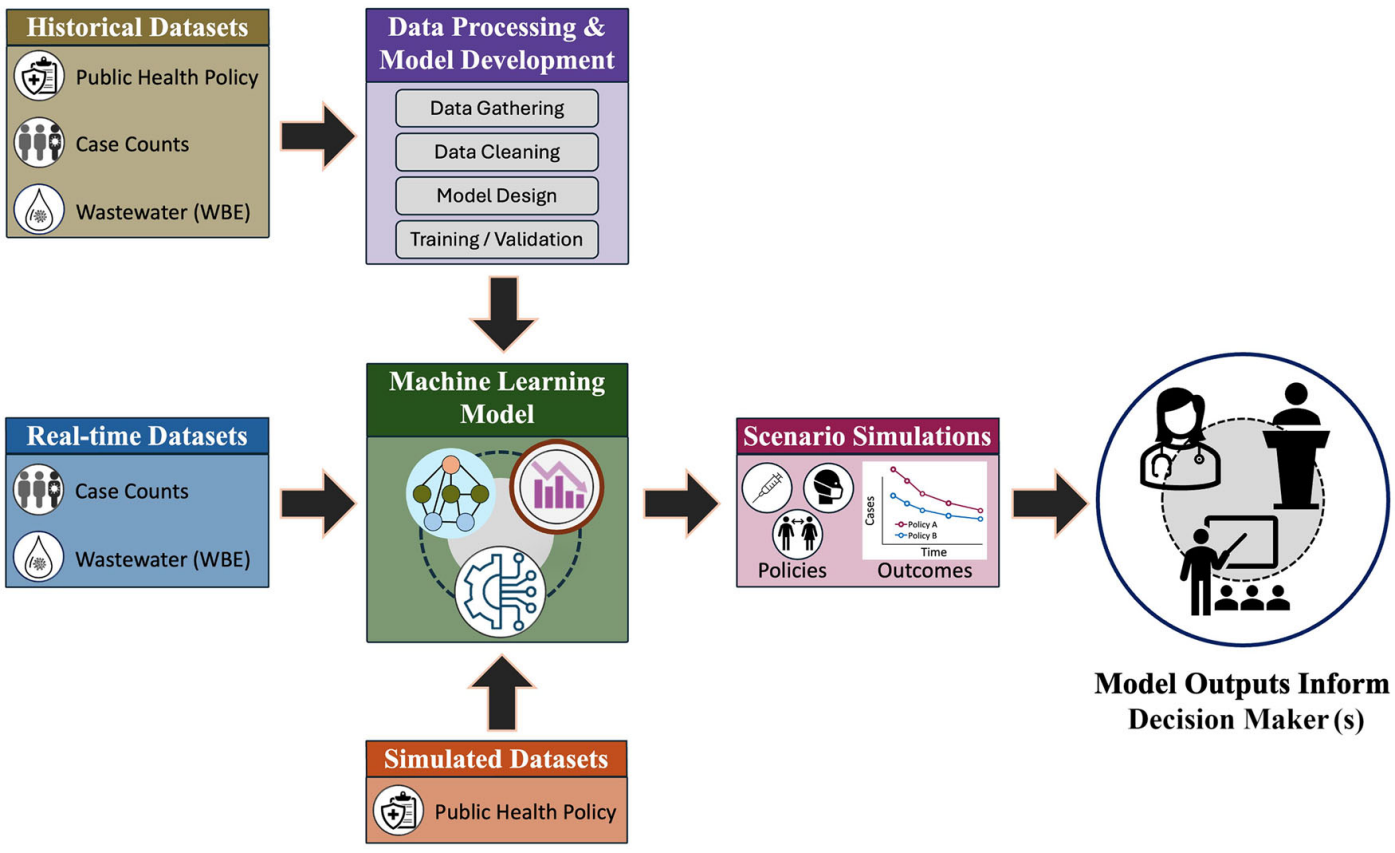
Workflow for data‐driven machine learning (ML) model and trend forecasting. Historical datasets are used to design, train, and validate the models, while real‐time and simulated datasets are employed to evaluate scenarios and predict the outcomes of health policy interventions. These predicted outcomes inform and support evidence‐based decision‐making.

Early efforts to model and forecast COVID‐19 infection trends primarily relied on susceptible‐exposed‐infectious‐recovered models and other conventional approaches (He et al., 2020; Mwalili et al., 2020). However, as L. Cao and Liu (2024) noted in their review of COVID‐19 modeling techniques, traditional methods (e.g., statistical and epidemiological models) lack cross‐domain and multidisciplinary modeling ability, limiting their predictive power. The advent of modern ML‐based methods, especially deep neural network‐based supervised learning, has enabled a new generation of data‐driven models designed to predict public health trends using increasingly available WBE datasets. Figure 1 illustrates the typical workflow for building such models: After preprocessing historical WBE data, case counts, and optionally, policy data, an ML model is designed, trained and validated. The trained model is then used to forecast public health trends using current or real‐time data. For decision‐making bodies, the value of these models extends beyond their capability to predict future trends (over days, weeks, or months) using current data. They also offer the potential to assess the impacts of hypothetical policy interventions, a capability that, to our knowledge, remains unexplored and largely underutilized in practice.

We enumerate a few representative modeling efforts and comment on their predictive capability, generalizability, and potential shortcomings. Y. Cao and Francis (2021) developed a vector autoregression (VAR) model to predict COVID‐19 case counts from wastewater SARS‐CoV‐2 viral load measurements from a wastewater treatment facility and determined that COVID‐19 cases peaked 3 weeks after viral loads spiked in wastewater. Zehnder et al. (2023) combined a support vector machine model with wastewater data and a hydraulic model of in‐sewer pathogen transport to identity COVID hotspots in the Netherlands. Galani et al. (2022) employed ML models to forecast COVID‐19 hospitalizations and intesive care unit (ICU) admissions using wastewater data from Athens, utilizing a Bayesian distributed‐lag nonlinear model to optimize the time lag, and presented two forecast models: a linear regression and an artificial neural network. Fazli and Shakeri (2023) explored deep learning models for COVID‐19 forecasting, comparing deep temporal convolutional networks (DeepTCN) and temporal fusion transformer (TFT) architectures using Biobot Analytics (Duvallet et al., 2022) and Oxford (Hale et al., 2023) datasets, demonstrating that among the independent variables, containment policies most significantly predicted COVID‐19 incidence followed by wastewater viral load.

The challenges associated with using ML models for public health forecasting can be broadly categorized into two key areas: datasets and models.

### 3.1 Datasets

The literature highlights several challenges related to data quality, including incomplete reporting and data inconsistencies. Many counties in the United States implemented wastewater monitoring only after the CDC launched its wastewater surveillance program, resulting in limited WBE data for some locations. Consequently, many models are trained on small datasets covering only a subset of the country. In some cases, data from a single wastewater treatment facility is used as the sole training source (Y. Cao & Francis, 2021; Galani et al., 2022). Multimodal data integration holds promise for improving prediction accuracy (L. Cao & Liu, 2024; Fazli & Shakeri, 2023), but effectively combining data from diverse sources remains a challenge. The complexity arises from differences in data formats, collection methodologies, and quality across independent projects. Addressing these challenges requires more robust techniques to integrate small‐scale datasets and reconcile inconsistencies in data records.

### 3.2 Models

(a) Data hungry: ML models generally require larger datasets for model training, which can lead to higher quality predictions. VAR models, for instance, capture linear relationships between multiple independent variables but demand substantial domain knowledge and high‐quality data. More advanced deep learning models such as DeepTCN and TFT can learn complex nonlinear relationships with minimal domain knowledge. However, these models require extensive data to prevent overfitting and are often difficult to interpret due to their “black box” nature. (b) Limited capability for predicting fine‐grained outcomes: Despite advances in COVID‐19 modeling, models struggle to capture the nuanced characteristics of the disease, such as varying severity levels and high proportion of asymptomatic infections (L. Cao & Liu, 2024; Wang et al., 2023; You et al., 2024). Current datasets primarily record positive case counts and aggregate wastewater pathogen concentrations but fail to distinguish between mild and severe infections or identify asymptomatic cases. This lack of granularity limits the models’ ability to provide detailed insights into disease progression and public health impacts.

## 4 PERSPECTIVES ON DATA‐DRIVEN DECISION‐MAKING IN PUBLIC HEALTH SAFETY

While data‐driven decision‐making is widely recognized, its integration into public health policy remains limited. Forecasting models trained on historical data play a critical role in risk analysis and policy formulation. For example, models that predict ICU admissions and hospitalizations using wastewater data (Galani et al., 2022) can serve as early warning systems, allowing healthcare facilities to prepare resources and implement interventions to mitigate disease spread. By forecasting pandemic trends, these models enable policymakers to assess the relative effectiveness of different interventions such as lockdowns, social distancing measures, vaccination campaigns, or economic incentives (Hale et al., 2023). However, despite their potential, data‐driven models have not fully been integrated in the decision‐making process, a challenge first explored in the pioneering work of Kahneman and Tversky (1979).

Forecasting models contribute to risk analysis by simulating various risk factors and evaluating the potential intervention outcomes, essentially modeling “what‐if” scenarios. This capability allows policymakers to assess an intervention's effectiveness relative to its costs while also quantifying the uncertainty associated with predictions. Kahneman's prospect theory suggests that forecasts influence risk perception. For example, if a model predicts a significant rise in cases, decision makers may perceive this as a potential loss, eliciting a stronger emotional response and urgency than a forecast suggesting a decline. This psychological bias, known as *loss aversion*, often motivates policymakers to prioritize actions that prevent negative outcomes, such as increased infection rates or deaths, over those that promote positive outcomes, such as economic relief measures.

**FIGURE 2:**
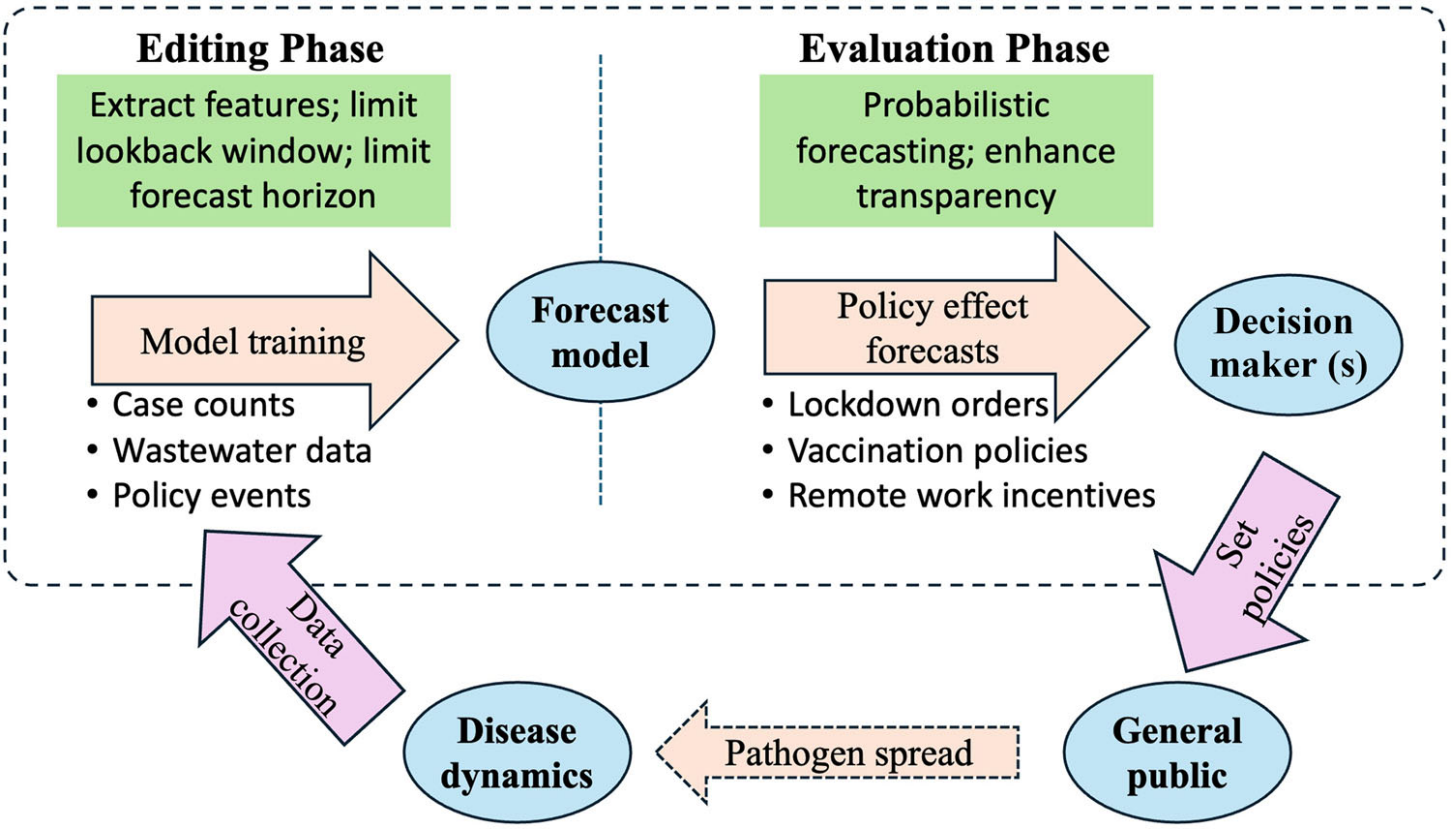
Framework for two‐stage data‐driven decision‐making in public health. In the *editing phase*, relevant data are filtered and prioritized to reduce model training time and enhance the generalizability and forecasting performance of ML models. In the *evaluation phase*, ML models support decision‐making through probabilistic forecasting of policy outcomes. The dashed boundary highlights the training process of the ML models and their integration into the decision‐making framework. Decision makers implement public health policies based on model insights, which in turn influence disease transmission within the community. The outcomes generate new data that can be used to recalibrate and improve future model forecasts.

Prospect theory proposes that human decision‐making occurs in a two‐stage process: the editing phase and the evaluation phase (Kahneman & Tversky, 1979). Figure 2 illustrates a framework for applying prospect theory in health‐related decision‐making, particularly in the context of data‐driven ML models. At a high level, the ML forecast model relies on data collection to obtain training data such as case counts, wastewater monitoring, and policy events (e.g., lockdowns). Decision makers leverage the model's forecasting capability and transparency to evaluate potential policy outcomes and effectively communicate decisions to the public.

### 4.1 Editing phase: Filtering and simplifying data

In the *editing phase*, decision makers filter and prioritize information before making final choices. This process does not involve altering data samples; rather, it involves selecting the most relevant datasets to reduce model training time, handle missing or noisy data, and improve generalizability. Given the increasing size and complexity of datasets across temporal and spatial dimensions, this phase allows decision makers to identify the most suitable training data for forecasting models. ML models mirror this process by preprocessing data and selecting key features that influence predictions. For instance, deep learning models like DeepTCN and TFT automatically extract meaningful patterns from data, learning relationships between daily case counts, wastewater viral load, and government response indices. These models simplify the forecasting problem by structuring it around the given dataset. Similarly, restricting the lookback window and forecasting horizon serves as another form of filtering, helping models refine predictions. In essence, deep learning models perform data abstraction, much like human decision makers, to generate insights.

### 4.2 Evaluation phase: Assessing risk and reward

In the *evaluation phase*, decision makers weigh the risks and benefits of different policy choices. ML models contribute to this phase through probabilistic forecasting, which provides decision makers with a range of possible outcomes. However, human decision‐making is not always rational. Kahneman and Tversky (1979) observed that people tend to be risk‐averse, often prioritizing loss avoidance over potential gains. For example, if a model predicts a range of possible case counts, policymakers may focus on the worst‐case scenario due to loss aversion, even if the “most likely” scenario is more optimistic. Some ML models, such as TFT (Fazli & Shakeri, 2023) provide some level of transparency in determining the relative importance of different input variables. By clarifying how inputs influence outputs, TFT models can help reduce uncertainty, making decision makers more confident in data‐driven recommendations. Enhancing transparency in forecasting models may encourage greater adoption of data‐driven approaches in public health decision‐making.

ML models, unlike human decision makers, are free from cognitive biases like risk and loss aversion. This objectivity makes them powerful tools for processing large datasets, identifying patterns, and generating unbiased insights. By analyzing historical and real‐time data, these models can detect trends and assess risks that might otherwise be overlooked. A key advantage is their ability to evaluate multiple scenarios objectively. While human decision makers may fixate on worst‐case outcomes, ML models provide probabilistic forecasts, enabling more balanced, data‐driven decisions. They can also integrate diverse data sources, such as epidemiological trends, socioeconomic factors, and mobility data, offering comprehensive risk assessments beyond the capability of current health surveillance and forecasting models. This is especially valuable in low‐resource settings, where ML models can expedite the analysis of WBE data, providing a cost‐effective approach for early outbreak detection (Kitajima et al., 2020). However, ML models are not without limitations. Their accuracy depends on data quality, and biases in training data can affect outcomes. Deep learning models, often viewed as “black boxes,” can also lack transparency, leading to skepticism in decision‐making. To maximize their potential, a hybrid approach is essential, combining the analytical power of artificial intelligence with human expertise to incorporate ethical, social, and policy considerations. Mittelstadt et al. (2016) further emphasizes the ethical imperative for transparency, fairness, and accountability in algorithmic decision‐making, especially in WBE, to prevent bias and uphold social justice in the use of artificial intelligence (AI) and ML for public health. Importantly, maximizing the explainability of AI systems can facilitate cooperation among stakeholders. Overall, ML enhances public health decision‐making by processing vast amounts of data without bias. However, success depends on high‐quality data, proper integration into policy frameworks, and balancing machine‐driven insights with human judgment.

## 5 CURRENT LIMITATIONS AND FUTURE DIRECTIONS

The adoption of data‐driven ML in public health monitoring faces significant challenges despite advances in ML and deep learning. A key barrier is the limited interpretability of many models due to their “black‐box” nature. As noted by Tonekaboni et al. (2019), clinicians—and by extension, public health professionals—require model explanations that align with domain‐specific reasoning. This necessitates that ML outputs be interpretable not only in general terms but also in the context of the data and decision‐making practices used by public health officials.

To be effective, AI systems must clearly communicate their informational basis, assumptions, and limitations. A degree of inaccuracy may be acceptable if the source of error is transparent and understandable. The research community has explored various methods to enhance explainability, including tools like SHAP and LIME (Salih et al., 2025). SHAP, for example, offers detailed feature attribution using game theory principles, considering all possible feature interactions. However, such tools can be computationally intensive and may present interpretability challenges for users without a strong statistical background, potentially limiting their practical utility in public health settings.

Data‐driven forecasting and decision‐making in public health benefit from (and often require) the explainability of ML models to ensure user trust and accountability. The broad acceptance of these AI models depends on their ability to align with established data metrics or uncover meaningful hidden patterns that are not immediately obvious. Advancing explainable AI in this context is essential for the continued integration of rich data sources into effective and informed public health strategies.

## CONFLICT OF INTEREST STATEMENT

The authors declare no conflicts of interest.

## Data Availability

Data sharing is not applicable to this article as no datasets were generated or analyzed during the current study.
